# Molecular Markers in Follicular and Oncocytic Thyroid Carcinomas: Clinical Application of Molecular Genetic Testing

**DOI:** 10.3390/curroncol31100441

**Published:** 2024-10-01

**Authors:** Alicia Belaiche, Grégoire B. Morand, Sena Turkdogan, Esther ShinHyun Kang, Véronique-Isabelle Forest, Marc P. Pusztaszeri, Michael P. Hier, Alex M. Mlynarek, Keith Richardson, Nader Sadeghi, Marco A. Mascarella, Sabrina D. Da Silva, Richard J. Payne

**Affiliations:** 1 Faculty of Medicine, McGill University, Montreal, QC H3G 2M1, Canada; alicia.belaiche@mail.mcgill.ca (A.B.);; 2 Department of Otolaryngology—Head and Neck Surgery, McGill University, Jewish General Hospital, Montreal, QC H3T 1E2, Canadasena.turkdogan@mail.mcgill.ca (S.T.);; 3 Department of Otorhinolaryngology—Head and Neck Surgery, University Hospital Zurich, 8091 Zurich, Switzerland; 4 Faculty of Medicine, University of Zurich, 8006 Zurich, Switzerland; 5 Department of Otolaryngology—Head and Neck Surgery, McGill University, Royal Victoria Hospital, Montreal, QC H4A 3J1, Canada; 6 Department of Pathology, McGill University, Jewish General Hospital, Montreal, QC H3T 1E2, Canada

**Keywords:** thyroid cancer, follicular thyroid carcinoma, oncocytic thyroid carcinoma, Hürthle cell carcinoma, molecular testing

## Abstract

Background: Oncocytic thyroid carcinoma (OTC) was previously considered a variant of follicular thyroid carcinoma (FTC) but has recently been reclassified as a separate form of thyroid cancer. This study aimed to demonstrate that FTC and OTC are fundamentally distinct entities that can potentially be differentiated preoperatively through cytology and/or molecular testing. Methods: A retrospective chart review of patients diagnosed with FTC and OTC operated upon at two university health centers from January 2016 to September 2023 (n = 3219) was conducted. Molecular testing results were correlated with histopathologic diagnosis. Results: Fifty patients met the inclusion criteria. FTC was identified in 27 (54.0%) patients, and OTC in 23 (46.0%) patients. Patients with OTC were older (61.8 years) than FTC patients (51.2 years) (*p* = 0.013). Moreover, aggressive tumors were found in 39.1% (9/23) of OTCs compared to 11.1% (3/27) of FTCs (*p* = 0.021). Amongst Bethesda category III and IV nodules, 17 out of 20 (85.0%) OTC cytology reports demonstrated an oncocytic subtype compared to only 5 out of 24 FTC cytology reports (20.8%) (*p* = 0.002). On molecular testing, the *EIF1AX* alteration was exclusively present in OTCs while the *PAX8/PPARy* and *PTEN* alterations were exclusively found in FTCs. Copy number alterations (*CNAs*) were found to be more prevalent in OTC (66.7%) compared to FTC (33.3%), and they were not indicative of tumor aggressiveness. Within the OTC group, all three patients who had a *TP53* alteration were diagnosed with aggressive cancer. Lastly, the OTCs exhibited a higher frequency of multiple alterations on molecular testing (66.7%) compared to FTCs (33.3%). Conclusion: To our knowledge, this is the largest study to date comparing the clinical application of abnormalities found on molecular testing for FTC and OTC. It further demonstrates the distinct clinicopathological and molecular characteristics of OTC.

## 1. Introduction

Thyroid cancer is the most common endocrine malignancy worldwide and has an increasing incidence among young adults, particularly women [1]. Thyroid cancer is divided into subtypes according to histology. Papillary thyroid carcinoma (PTC) is the most common, and rarer subtypes of well-differentiated thyroid cancer include follicular thyroid carcinoma (FTC) and oncocytic thyroid carcinoma (OTC), which make up 6–10% and 3% of all thyroid cancers, respectively [2,3]. OTC was previously considered a variant of FTC and often called Hürthle cell carcinoma. However, since 2017, the World Health Organization (WHO) has recognized it as a distinct entity among thyroid cancers, given its distinct clinical, morphological, and molecular features [4].

With the advent of molecular testing, clinicians have been encouraged to supplement their assessment of thyroid nodules with molecular testing in an effort to avoid diagnostic surgery, especially two-stage surgery (diagnostic hemi-thyroidectomy followed by completion thyroidectomy) [5]. Molecular testing enables the identification of molecular alterations (e.g., somatic DNA variations, gene fusions, copy number alterations) that are present in a particular lesion using next-generation sequencing. Different molecular tests such as *ThyGenX*, *ThyGeNEXT*, and *ThyroSeq V3* have been developed. *ThyGenX* includes a relatively small panel of genetic alterations frequently observed in PTC and other thyroid neoplasms. *ThyGeNEXT* and *ThyroSeq V3* were later developed and expanded to cover a broader range of molecular alterations, improving diagnostic accuracy [6,7,8].

The identification of specific alterations and molecular profiles by molecular testing greatly increases the likelihood of finding an abnormality or disease, and it can be used as an indicator to determine the risk of malignancy, the most probable type of thyroid cancer, or the potential aggressiveness of a nodule [9]. Moreover, when such biomarkers are absent, malignancy is less likely, and patients can avoid exploratory or extensive thyroid surgery. In particular, *BRAF*-like alterations correlate with malignancy and advanced stages of the disease [10]. In contrast, *RAS*-like alterations are often associated with less aggressive malignancies and indolent tumors such as follicular adenoma and NIFTP [11,12]. Non-*BRAF*-non-*RAS* (NBNR) tumors represent a novel subtype of thyroid tumors, often associated with oncocytic features [9].

The pathological findings and biomarkers have been extensively studied for PTC. However, the application of molecular findings in the clinical setting of FTC and OTC remains limited due to the rarity of these tumors. Additionally, due to the historical classification of OTC as a subtype of FTC, there is a paucity of studies directly addressing OTC and its molecular profile. Thus, we performed a retrospective review of patients with OTC and FTC with preoperative molecular testing in high-volume tertiary centers. The study objective was to demonstrate that OTC and FTC are fundamentally distinct entities that can potentially be differentiated preoperatively through cytology and/or molecular testing.

## 2. Materials and Methods

### 2.1. Study Design and Population

This study was a retrospective chart review of patients who received surgical treatment for thyroid nodules at two university health centers from January 2016 to September 2023. Eligibility criteria included previously untreated patients with FTC and OTC on their final surgical pathology, without any distant metastasis at the time of diagnosis (M0), who underwent a thyroid fine-needle aspiration (FNA) biopsy and received pre-operative molecular testing.

### 2.2. Molecular Testing

Three different types of molecular tests were used pre-operatively: *ThyGenX*, *ThyGeNEXT*, and *ThyroSeq V3* [6,7,8]. Three tests were studied to optimize the data collected, given the relative rarity of FTC and OTC and the relatively recent introduction of molecular testing into clinical practice. These molecular tests use next-generation sequencing to provide specific gene variants with their associated risk of malignancy. The choice of molecular test was determined by the patient after a discussion with the physician about their clinical picture and the test accessibility. The physician did not know the diagnosis before molecular test selection.

### 2.3. Operative Approach and Histopathological Analysis

Patients underwent either total thyroidectomy or hemi-thyroidectomy with a sentinel lymph node biopsy and central neck dissection when required. Histological diagnosis, tumor size, margins, lymph-vascular invasion, perineural invasion, extra-capsular extension, and extra-thyroidal extension were then examined by experienced thyroid pathologists at our institution. The thyroid FNA reports were classified according to the Bethesda System for Reporting Thyroid Cytopathology, 2018, by thyroid pathologists preoperatively [13]. The tumors were classified according to the 2017 WHO classification of thyroid tumors by thyroid pathologists after surgical removal of the tumor [4].

Aggressive tumors were defined as having at least one of the following features: extrathyroidal extension (ETE), presence of poorly differentiated thyroid carcinoma (PDTC), presence of high-grade features such as tumor necrosis and mitotic activity ≥5 per 2mm^2^, extensive angioinvasion defined as 4 or more foci of invasion, and/or widely invasive according to the WHO tumor classification. PDTC diagnosis was based on the Turin consensus criteria: (i) presence of a solid/trabecular/insular pattern of growth in a tumor diagnosed as malignant based on invasive properties; (ii) absence of conventional nuclear features of PTC; (iii) presence of at least one of the following: convoluted nuclei, mitotic count ≥3 per 2 mm^2^, tumor necrosis.

### 2.4. Statistical Analysis

Statistical analyses of associations between categorical variables were performed by the Fisher’s exact test and for continuous variables the non-parametric Mann–Whitney U test (with significance set for *p* < 0.05). All analyses were performed by a statistician, using the statistical software package STATA-13 (STATA Corporation, College Station, TX, USA).

## 3. Results

### 3.1. Clinicopathological Findings

**Figure 1:**
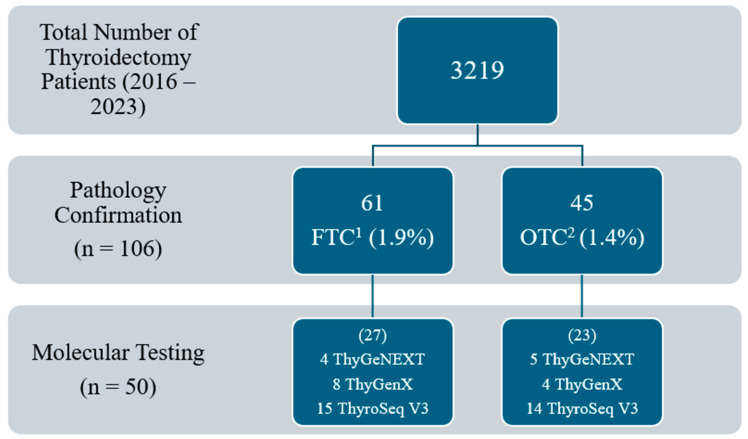
Total number of patients meeting inclusion criteria. ^1^ FTC: follicular thyroid carcinoma. ^2^ OTC: oncocytic thyroid carcinoma.

There was a total of 3219 patients with thyroid nodules who underwent thyroid surgery between January 2016 and September 2023. Of the 3219 patients, 50 (1.6%) met the inclusion criteria of which 27 out of 50 (54.0%) had FTC and 23 out of 50 (46.0%) had OTC (Figure 1).

Table 1 describes the clinicopathological characteristics of the study population. Of the 27 FTC patients, 23 (85.2%) were female and 4 (14.8%) were male with an average age of 51.1 years (SD ± 15.4). Of the 23 OTC patients, 15 (65.2%) were female and 8 (34.8%) were male with an average age of 61.8 years (SD ± 13.2). In terms of surgical management, 23 FTC patients (85.2%) underwent hemi-thyroidectomy and 4 (14.8%) underwent total thyroidectomy. In the OTC group, there were 15 hemi-thyroidectomies (65.2%) and 8 total thyroidectomies (34.8%).

Regarding pathological findings, in FTC patients, the mean nodule size was 3.0 cm: 6 nodules (22.2%) were <2 cm, 11 (40.7%) were ≥2–<3 cm, 2 (7.4%) were ≥3–<4 cm, and 8 (29.6%) were ≥4 cm. Twenty-two FTCs (81.5%) were minimally invasive, two FTC cases (7.4%) were encapsulated angioinvasive, and three FTC cases (11.1%) were widely invasive. One of the widely invasive FTC cases showed high-grade features with extensive angioinvasion. Another widely invasive FTC tumor showed 50% of the area with PDTC and 20% with anaplastic thyroid carcinoma and was associated with extrathyroidal extension and extensive angioinvasion (Table 1).

In OTC patients, the mean nodule size was 3.5 cm: five nodules (21.7%) were <2 cm, four (17.4%) were ≥2–<3 cm, six (26.1%) were ≥3–<4 cm, and eight (34.8%) were ≥4 cm. Thirteen OTC cases (56.5%) were minimally invasive (including one with a PDTC component of 40%), seven OTC cases (30.4%) were encapsulated angioinvasive (including one with a PDTC component of less than 30% and extensive angioinvasion, one with a PDTC component of 5–10%, and one with tumor necrosis and extensive angioinvasion), and three OTC cases (13.0%) were widely invasive (including one with a 40–50% PDTC component and extensive angioinvasion) (Table 1). One aggressive OTC tumor exhibited extrathyroidal extension and another one showed extensive angioinvasion.

In terms of cytology results, in the FTC patients, 8 nodules (29.6%) were Bethesda category III (with only 1 out of 8 being oncocytic subtype), 16 (59.3%) were category IV (with only 4 out of 16 being oncocytic subtype), 2 (7.4%) were category V, and 1 (3.7%) was category VI. In OTC patients, 8 nodules (34.8%) were category III (with 6 out of 8 being oncocytic subtype), 12 (52.2%) were category IV (with 11 out of 12 being oncocytic subtype), 2 (8.7%) were category V, and 1 (4.3%) was category VI. Overall, amongst Bethesda category III and IV nodules, within OTC, 17 out of 20 (85.0%) cytology reports demonstrated an oncocytic subtype compared to only 5 out of 24 cytology reports (20.8%) in FTC (Table 1).

OTC, with 9 out of 23 cases (39.1%) being aggressive, demonstrated a higher level of aggressiveness compared to FTC, where only 3 out of 27 cases (11.1%) were aggressive tumors. This difference was statistically significant (*p* = 0.021). Additionally, the mean age was statistically significantly higher in patients diagnosed with OTC (61.8 years) compared to FTC (51.1 years) (*p* = 0.013) (Table 1). We found that the three widely invasive FTC patients were female (100.0%), whereas the three widely invasive OTC patients were male (100.0%). Furthermore, there could be a tendency for OTC to be more prevalent in males given that 8 out of 23 patients with OTC (34.8%) were males while 4 out of 27 patients with FTC (14.8%) were males (*p* = 0.099).

### 3.2. Molecular Findings

Molecular testing was performed using *ThyroSeq V3* in 29 patients, *ThyGeNEXT* in 9 patients, and *ThyGenX* in 12 patients. Of the 50 patients whose biopsy samples were submitted for molecular testing, 33 (66.0%) had an abnormality found on specific alterations and/or molecular profile; 28/33 (84.8%) were *ThyroSeq V3*, 4/33 (12.1%) were *ThyGeNEXT*, and 1/33 (3.0%) was *ThyGenX*. The molecular findings in aggressive and non-aggressive FTCs and OTCs are described in Table 2.

Molecular markers associated with FTC included *PAX8/PPARγ* fusion (n = 4), copy number alterations (*CNAs*) (n = 4), *NRAS* (n = 3), *PTEN* (n = 2), gene expression profile (*GEP*) (n = 2), *HRAS* (n = 1), *TERT* (n = 1), *TP53* (n = 1), *DICER1* (n = 1), *THADA-IGF2BP3* (n = 1), and *BRAF p.R462I* (n = 1). Molecular markers detected in OTC comprised *CNAs* (n = 8), *EIF1AX* (n = 4), *TERT* (n = 4), *TP53* (n = 3), *GEP* (n = 2), *HRAS* (n = 2), *NRAS* (n = 1), *PIK3CA* (n = 1), and *DICER1* (n = 1).

Out of the 17 abnormal molecular results in FTC, 4 (23.5%) exhibited a *PAX8/PPARγ* alteration and 2 (11.8%) showed a *PTEN* alteration, markers that were both exclusive to FTC in our study. Out of the 16 abnormal molecular results in OTC, 4 (25.0%) harbored an *EIF1AX* alteration, which was unique to OTC (Table 2). Hence, while the presence of the *PAX8/PPARγ* and *PTEN* alterations on molecular testing suggested the presence of FTC over OTC, the detection of *EIF1AX* increased the likelihood of OTC compared to FTC.

*RAS* alterations were present in both FTC and OTC. Indeed, 4 out of 17 FTC abnormal molecular test results (23.5%) and 3 out of 16 OTC abnormal molecular test results (18.8%) harbored an *RAS* alteration on molecular testing. Molecular markers that are commonly aggressive such as *TERT* and *TP53* were more prevalent in OTC compared to FTC. Indeed, *TERT* promoter alterations were present in four OTCs versus one FTC. *TP53* alterations were present in three OTCs versus one FTC. Within the OTC subgroup, all three patients with *TP53* alterations were diagnosed with aggressive thyroid cancer. *GEPs*, *DICER1*, and *THADA-IGF2BP3* alterations were all infrequently observed and uniformly identified in the non-aggressive tumors (Table 2).

Copy number alterations (*CNAs*) were present in 12 out of 33 (36.4%) abnormal molecular results, indicating their significant prevalence in OTC and FTC. However, *CNAs* were found to be more frequent in OTC compared to FTC as 8 out of 12 patients (66.7%) with *CNAs* were diagnosed with OTC, whereas 4 out of 12 (33.3%) were diagnosed with FTC. Additionally, the presence of *CNAs* on molecular testing was equally distributed between aggressive and non-aggressive tumors (Table 2).

A higher incidence of multiple alterations was observed in OTC compared to FTC. Amongst the nine patients who exhibited multiple genetic changes after molecular testing, six out of nine (66.7%) were diagnosed with OTC, and three out of nine (33.3%) were diagnosed with FTC. Notably, both cases of aggressive FTC with abnormal molecular tests showed three alterations (Table 2).

One FTC tumor with a *TP53* alteration, one OTC tumor with *TERT* promoter + *PIK3CA* + *EIF1AX* alterations, and one OTC tumor with a *TERT* promoter alteration were classified as non-aggressive on surgical pathology. It could be due to being detected early in their pathogenesis and clinical course. At the time of surgical resection, they had, respectively, minimal tumor capsular invasion and focal lymph-vascular invasion, minimal tumor capsular invasion and partial tumor encapsulation, and minimal tumor capsular invasion with margins focally involved by carcinoma.

Regarding the performance of the molecular tests, *ThyroSeq V3* had a detection rate of 96.6% (28/29), which is significantly higher than the rates of 44.4% (4/9) for *ThygeNEXT* and 8.3% (1/12) for *ThyGenX*. Additionally, *ThyroSeq V3*, in contrast to *ThygeNEXT* and *ThyGenX*, consistently identified abnormalities in aggressive tumors, demonstrating reliability in informing pre-operative decision-making.

## 4. Discussion

Recent literature has increasingly recognized OTC as an independent entity within the spectrum of malignant thyroid neoplasms. In this context, it has become crucial to define the clinicopathological and molecular characteristics of OTC compared to FTC, especially considering that OTC has been classified and studied as a subtype of FTC up until recently and that both cancers are relatively rare.

Our pathological findings demonstrate a higher proportion of aggressive tumors in OTC compared to FTC. The existing literature specifically on OTC is limited but generally characterizes it as exhibiting more aggressive behavior compared to other types of differentiated thyroid cancers. Available studies report a worse prognosis and greater incidence of distant metastases in patients presenting with widely invasive OTC and/or oncocytic PDTC [14,15]. In a cohort of 111 patients, Matsuura et al. found that OTC patients were more likely to exhibit extensive vascular invasion and to be older than 55 years old, compared to FTC patients [16]. This latter finding regarding older age in OTC patients was also statistically significant in our study. In terms of sex-specific findings, male sex has been associated with widely invasive OTC in the literature [17,18]. Within our patient cohort, all cases of widely invasive OTC were observed in males (three out of three, 100%), whereas all cases of widely invasive FTC were observed in females (three out of three, 100%).

In the Bethesda system for reporting thyroid cytopathology, FNA specimens that are suspicious for an oncocytic neoplasm have been traditionally distinguished from those suspicious for a non-oncocytic follicular neoplasm because there is a striking morphologic difference between these two cytologic patterns, which raises different diagnostic considerations. Indeed, the oncocyte, which is defined morphologically as a thyroid follicular cell with an abundance of finely granular cytoplasm that reflects an excessive number of mitochondria, is morphologically distinct from its non-oncocytic counterpart and can be readily identified on cytology. Accordingly, our results show that cytology results may serve as an adjunct in the pre-operative distinction between OTC and FTC. Indeed, for Bethesda category III and IV tumors, we found a significantly higher proportion of oncocytic subcategories in OTC compared to FTC (*p* = 0.002). Furthermore, there is data to suggest that follicular and oncocytic carcinomas are genetically different neoplasms [14].

Molecularly, the initiation of FTC is believed to stem from *RAS* alterations, *PAX8/PPARγ* fusions, and *PI3K/AKT* pathway activation by either *PI3KCA*, *AKT1*, or *PTEN* alterations. Transition to higher grade or undifferentiated tumors is often the result of additional genetic or epigenetic changes such as *TERT* promoter alterations, *TP53* alterations, or increased *CNAs* [19]. On the other hand, OTC sets itself as a distinct type of thyroid cancer notably through the presence of mitochondrial DNA variations and widespread genomic chromosomal loss of heterozygosity [20]. Other associated genetic changes include *RAS*, *EIF1AX*, *DAXX*, *NF1*, *TP53*, or *TERT* promoter alterations [21].

In our study, *PAX8/PPARγ* fusions were exclusively found in FTCs. Nikiforova et al. found a similar result in a study investigating *PAX8/PPARy* detected through RT-PCR in FTC and OTC [22]. It was also suggested that these alterations are absent in OTC, which aligns with our findings [23]. *PAX8/PPARγ* alterations are described in the literature as a characteristic marker of FTC [24], along with the encapsulated follicular variant of PTC [25]. Hlozek et al. described the malignancy risk associated with a *PAX8/PPARγ* fine needle aspiration cytology finding as ranging between 84.6% and 95% [24], while Armstrong et al. found a malignancy risk of 100% [25]. However, evidence is lacking regarding its potential prognostic value, and our data does not appear to demonstrate a correlation with tumor aggressiveness [26]. RAS isoform alterations are another well-known somatic DNA variation in FTC and, to a lesser extent, in OTC; findings that were observed in our study [17,23,27]. Additionally, our results indicate the exclusive detection of *EIF1AX* in OTC and *PTEN* in FTC. This may suggest an increased prevalence of these genetic abnormalities in these respective malignancies, potentially helping to distinguish between the two cancer types. Indeed, in a comprehensive genomic characterization of 56 primary OTC tumors, Ganly et al. have found a high occurrence of *EIF1AX* alterations in OTC, with a prevalence comparable to that observed in PDTC and anaplastic thyroid cancers [28]. Regarding the *PTEN* molecular marker, additional studies remain necessary to confirm the observed trend as we found two studies that reported *PTEN* detection in OTC [28,29].

A prominent molecular marker in our OTC data and, to a lesser extent, in our FTC data, is copy number alterations (*CNAs*). In 2012, Corver et al. reported widespread DNA copy number alterations, typically leading to near-complete genome haploidization, as a likely genetic mechanism of OTC [30]. More recently, two studies that used whole exome sequencing to analyze a large number of OTCs reported *CNAs* in the form of genome haploidization in more than half of OTCs, with additional tumors showing focal *CNA* [2,28]. These studies suggested that *CNAs* represent a genomic hallmark of OTC, even though they also occur at a lower frequency in FTC and benign oncocytic adenoma [31]. Additionally, in our study, *CNAs* were equally distributed between aggressive and non-aggressive tumors. Therefore, they were not indicative of tumor aggressiveness, whether present alone or with another molecular marker.

*TP53* and *TERT* promoter alterations have been associated with invasive and undifferentiated thyroid carcinomas. The results of our study demonstrate a higher prevalence of these two aggressive markers in OTC compared to FTC, which aligns with the clinical course of these cancers. Moreover, *TP53* alterations were exclusively detected in aggressive OTCs, suggesting a strong association between *TP53* alterations and aggressive OTC. In a case cohort of 12 clinically aggressive OTCs, Wei et al. observed that 42% of the tumors harbored a *TP53* alteration [29]. *TERT* promoter alterations, on the other hand, have been reported as frequent in OTC, particularly in widely invasive cases [17,28].

In our study, *DICER1* and *THADA-IGF2BP3* alterations were infrequent and only detected in non-aggressive tumors. In the literature, they have been recognized as low-risk markers, often occurring in follicular adenomas, NIFTPs, or low-risk PTC [32,33,34]. In the case of *BRAF V600E* alterations, they are classically found in PTC. The literature suggests that they are absent in OTC, which was observed in our study [28,35].

Abnormalities were identified in 66.0% of the cases tested by molecular testing. Of the three molecular tests, *ThyroSeq V3* was found to be superior in its ability to identify molecular abnormalities, followed by *ThyGeNEXT*, then *ThyGenX*. However, this discrepancy is well explained by the broader-spectrum gene panel used in *ThyroSeq V3* and *ThyGeNEXT*, which tests for more than double the number of variations detected by *ThyGenX* [6,7,8]. Moreover, while earlier versions of molecular testing were not ideal for identifying OTC, the expanded panels including copy number alterations in the current molecular tests, such as *ThyroSeq V3*, have made pre-operative diagnosis of OTC more promising [8].

Our study has some limitations. First, as this was a retrospective analysis, pathologists were not specifically blinded to the molecular results of FNA testing at the time of surgical pathology evaluation. Secondly, our study was limited by the relatively small sample size. As molecular testing is still in its early stages, future larger-scale studies will be required to confirm our observations. It is important to note, however, that FTC and OTC are uncommon, and our numbers are reflective of their usual prevalence. Furthermore, as the cases were collected only from local teaching hospitals, results may have been influenced by a geographical bias. Thirdly, three different molecular tests were used, and the diagnostic performance of these tests was variable.

## 5. Conclusions

To our knowledge, this is the largest study to date comparing the clinical application of abnormalities found in commercially available molecular testing for follicular and oncocytic thyroid carcinomas. It makes a point in further demonstrating the distinct clinicopathological and molecular characteristics of OTC, which likely develops via separate molecular events. According to our results, OTC patients had more aggressive tumors, which further justifies the importance of better defining their molecular profile to improve preoperative decision-making. Cytology results revealed considerable potential in differentiating OTC from FTC preoperatively. Together, cytology results and molecular testing offer a promising avenue for developing a more tailored management approach for this distinct thyroid neoplasm.

## Figures and Tables

**Table 1:** Clinicopathological characteristics of study population.

Variables	FTC ^1^ *n* (%)	OTC ^2^ *n* (%)	*p*-Value
Number of patients	27	23	N/A
Age (years)	51.1 (SD ± 15.4)	61.8 (SD ± 13.2)	0.013 *
Sex Female Male	23 (85.2%) 4 (14.8%)	15 (65.2%) 8 (34.8%)	0.099 **
Type of surgery Hemi-thyroidectomy Total thyroidectomy	23 (85.2%) 4 (14.8%)	15 (65.2%) 8 (34.8%)	0.099 **
Bethesda classification III IV V VI	8 (29.6%) 16 (59.3%) 2 (7.4%) 1 (3.7%)	8 (34.8%) 12 (52.2%) 2 (8.7%) 1 (4.3%)	N/A
Bethesda III and IV Subcategory Oncocytic Not otherwise specified	5 (20.8%) 19 (79.2%)	17 (85.0%) 3 (15.0%)	0.002 **
Tumor size <2 cm ≥2–<3 cm ≥3–<4 cm ≥4 cm	6 (22.2%) 11 (40.7%) 2 (7.4%) 8 (29.6%)	5 (21.7%) 4 (17.4%) 6 (26.1%) 8 (34.8%)	N/A
Invasion Minimally invasive Angioinvasive Widely invasive	22 (81.5%) 2 (7.4%) 3 (11.1%)	13 (56.5%) 7 (30.4%) 3 (13.0%)	N/A
Presence of aggressive features ^3^ Minimally invasive Angioinvasive Widely invasive	0 (0.0%) 0 (0.0%) 2 (7.4%)	1 (4.3%) 3 (13.0%) 1 (4.3%)	N/A
Tumor aggressiveness ^4^ Aggressive Non-aggressive	3 (11.1%) 24 (88.9%)	9 (39.1%) 14 (60.9%)	0.021 **

* Mann–Whitney U test; ** Fisher’s exact test. ^1^ FTC: follicular thyroid carcinoma. ^2^ OTC: oncocytic thyroid carcinoma. ^3^ Aggressive features: Presence of poorly differentiated thyroid carcinoma (PDTC) as defined by the Turin criteria and/or presence of high-grade features such as tumor necrosis and/or mitotic activity ≥5 per 2 mm^2^. ^4^ Aggressive tumor: defined as having at least one of the following features: Presence of poorly differentiated thyroid carcinoma as defined by the Turin criteria. Presence of high-grade features such as tumor necrosis and mitotic activity ≥5 per 2 mm^2^. Extensive angioinvasion defined as four or more foci of invasion. Extrathyroidal extension. Widely invasive as per WHO tumor classification.

**Table 2:** Molecular testing results of aggressive ^1^ and non-aggressive tumors classified as follicular thyroid carcinoma (FTC) and oncocytic thyroid carcinoma (OTC).

Tumor Aggressiveness	Molecular Test	FTC Molecular Result	OTC Molecular Result
Aggressive	*ThyroSeq V3*	*CNA + Pax8/PPARy* *TERT + HRAS + CNA*	*TP53 + EIF1AX* *TP53 + EIF1AX + NRAS + CNA* *TP53 + CNA* *CNA* *CNA*
	*ThyGenX*	No alteration	No alteration
	*ThygeNEXT*	-	*TERT* *TERT* No alteration
Non-Aggressive	*ThyroSeq V3*	*Pax8/PPARy* *Pax8/PPARy* *PTEN* *PTEN* *CNA* *CNA* *NRAS* *NRAS + GEP* *GEP* *DICER1* *THADA-IGF2BP3* *BRAF p.R462I* *TP53*	*EIF1AX + TERT + PIK3CA* *EIF1AX* *HRAS + GEP* *HRAS + GEP + CNA* *CNA* *CNA* *CNA* *DICER1* No alteration
	*ThyGenX*	*NRAS* No alteration x6	No alteration × 3
	*ThygeNEXT*	*Pax8/PPARy* No alteration x3	*TERT* No alteration

^1^ Aggressive tumor: defined as having at least one of the following features: Presence of poorly differentiated thyroid carcinoma as defined by the Turin criteria. Presence of high-grade features such as tumor necrosis and mitotic activity ≥5 per 2 mm^2^. Extensive angioinvasion defined as four or more foci of invasion. Extrathyroidal extension. Widely invasive as per WHO tumor classification.

## Data Availability

The data presented in this study are available upon request from the corresponding author. The data are not publicly available due to the ethics approval agreement.

## References

[1] Miranda-Filho A., Lortet-Tieulent J., Bray F., Cao B., Franceschi S., Vaccarella S., Dal Maso L. (2021). Thyroid Cancer Incidence Trends by Histology in 25 Countries: A Population-Based Study. Lancet Diabetes Endocrinol..

[2] Gopal R.K., Kübler K., Calvo S.E., Polak P., Livitz D., Rosebrock D., Sadow P.M., Campbell B., Donovan S.E., Amin S. (2018). Widespread Chromosomal Losses and Mitochondrial DNA Alterations as Genetic Drivers in Hürthle Cell Carcinoma. Cancer Cell.

[3] McHenry C.R., Phitayakorn R. (2011). Follicular Adenoma and Carcinoma of the Thyroid Gland. Oncol..

[4] Bai Y., Kakudo K., Jung C.K. (2020). Updates in the Pathologic Classification of Thyroid Neoplasms: A Review of the World Health Organization Classification. Endocrinol. Metab..

[5] Cibas E.S., Ali S.Z. (2017). The 2017 Bethesda System for Reporting Thyroid Cytopathology. J. Am. Soc. Cytopathol..

[6] Zhang M., Lin O. (2016). Molecular Testing of Thyroid Nodules: A Review of Current Available Tests for Fine-Needle Aspiration Specimens. Arch. Pathol. Lab. Med..

[7] Ablordeppey K.K., Timmaraju V.A., Song-Yang J.W., Yaqoob S., Narick C., Mireskandari A., Finkelstein S.D., Kumar G. (2020). Development and Analytical Validation of an Expanded Mutation Detection Panel for Next-Generation Sequencing of Thyroid Nodule Aspirates. J. Mol. Diagn..

[8] Nikiforov Y.E., Baloch Z.W. (2019). Clinical Validation of the ThyroSeq v3 Genomic Classifier in Thyroid Nodules with Indeterminate FNA Cytology. Cancer Cytopathol..

[9] Morand G.B., Tessler I., Noik M., Krasner J., Yamin T., Pusztaszeri M.P., Avior G., Payne R.J. (2024). Molecular Profiling for Bethesda III to VI Nodules: Results of a Multicenter International Retrospective Study. Endocr. Pract..

[10] Krasner J.R., Alyouha N., Pusztaszeri M., Forest V.-I., Hier M.P., Avior G., Payne R.J. (2019). Molecular Mutations as a Possible Factor for Determining Extent of Thyroid Surgery. J. Otolaryngol. -Head Neck Surg..

[11] Mascarella M.A., Peeva M., Forest V.-I., Pusztaszeri M.P., Avior G., Tamilia M., Mlynarek A.M., Hier M.P., Payne R.J. (2022). Association of Bethesda Category and Molecular Mutation in Patients Undergoing Thyroidectomy. Clin. Otolaryngol..

[12] Medici M., Kwong N., Angell T.E., Marqusee E., Kim M.I., Frates M.C., Benson C.B., Cibas E.S., Barletta J.A., Krane J.F. (2015). The Variable Phenotype and Low-Risk Nature of RAS-Positive Thyroid Nodules. BMC Med..

[13] Ali S., Cibas E. (2018). The Bethesda System for Reporting Thyroid Cytopathology: Definitions, Criteria and Explanatory Notes.

[14] McFadden D.G., Sadow P.M. (2021). Genetics, Diagnosis, and Management of Hürthle Cell Thyroid Neoplasms. Front. Endocrinol..

[15] Xu B., Lubin D.J., Dogan S., Ghossein R.A., Viswanathan K. (2023). Significance of Oncocytic Features in Poorly Differentiated Thyroid Carcinoma—A Bi-Institutional Experience. Virchows Arch..

[16] Matsuura D., Yuan A., Wang L., Ranganath R., Adilbay D., Harries V., Patel S., Tuttle M., Xu B., Ghossein R. (2022). Follicular and Hurthle Cell Carcinoma: Comparison of Clinicopathological Features and Clinical Outcomes. Thyroid.

[17] Kure S., Ohashi R. (2021). Thyroid Hürthle Cell Carcinoma: Clinical, Pathological, and Molecular Features. Cancers.

[18] Chindris A.-M., Casler J.D., Bernet V.J., Rivera M., Thomas C., Kachergus J.M., Necela B.M., Hay I.D., Westphal S.A., Grant C.S. (2015). Clinical and Molecular Features of Hürthle Cell Carcinoma of the Thyroid. J. Clin. Endocrinol. Metab..

[19] Alzahrani A.S. (2023). Clinical Use of Molecular Data in Thyroid Nodules and Cancer. J. Clin. Endocrinol. Metab..

[20] Bischoff L.A., Ganly I., Fugazzola L., Buczek E., Faquin W.C., Haugen B.R., McIver B., McMullen C.P., Newbold K., Rocke D.J. (2024). Molecular Alterations and Comprehensive Clinical Management of Oncocytic Thyroid Carcinoma: A Review and Multidisciplinary 2023 Update. JAMA Otolaryngol. -Head Neck Surg..

[21] Rajab M., Payne R.J., Forest V.-I., Pusztaszeri M. (2022). Molecular Testing for Thyroid Nodules: The Experience at McGill University Teaching Hospitals in Canada. Cancers.

[22] Nikiforova M.N., Lynch R.A., Biddinger P.W., Alexander E.K., Dorn G.W., Tallini G., Kroll T.G., Nikiforov Y.E. (2003). RAS Point Mutations and PAX8-PPARγ Rearrangement in Thyroid Tumors: Evidence for Distinct Molecular Pathways in Thyroid Follicular Carcinoma. J. Clin. Endocrinol. Metab..

[23] Wong K.S., Angell T.E., Barletta J.A., Krane J.F. (2021). Hürthle Cell Lesions of the Thyroid: Progress Made and Challenges Remaining. Cancer Cytopathol..

[24] Hlozek J., Pekova B., Rotnágl J., Holý R., Astl J. (2022). Genetic Changes in Thyroid Cancers and the Importance of Their Preoperative Detection in Relation to the General Treatment and Determination of the Extent of Surgical Intervention—A Review. Biomedicines.

[25] Armstrong M.J., Yang H., Yip L., Ohori N.P., McCoy K.L., Stang M.T., Hodak S.P., Nikiforova M.N., Carty S.E., Nikiforov Y.E. (2014). PAX8/PPARγ Rearrangement in Thyroid Nodules Predicts Follicular-Pattern Carcinomas, in Particular the Encapsulated Follicular Variant of Papillary Carcinoma. Thyroid.

[26] Song Y.S., Lim J.A., Park Y.J. (2015). Mutation Profile of Well-Differentiated Thyroid Cancer in Asians. Endocrinol. Metab..

[27] Acuña-Ruiz A., Carrasco-López C., Santisteban P. (2023). Genomic and Epigenomic Profile of Thyroid Cancer. Best Pract. Res. Clin. Endocrinol. Metab..

[28] Ganly I., Makarov V., Deraje S., Dong Y., Reznik E., Seshan V., Nanjangud G., Eng S., Bose P., Kuo F. (2018). Integrated Genomic Analysis of Hürthle Cell Cancer Reveals Oncogenic Drivers, Recurrent Mitochondrial Mutations, and Unique Chromosomal Landscapes. Cancer Cell.

[29] Wei S., LiVolsi V.A., Montone K.T., Morrissette J.J.D., Baloch Z.W. (2015). PTEN and TP53 Mutations in Oncocytic Follicular Carcinoma. Endocr. Pathol..

[30] Corver W.E., Ruano D., Weijers K., den Hartog W.C.E., van Nieuwenhuizen M.P., de Miranda N., van Eijk R., Middeldorp A., Jordanova E.S., Oosting J. (2012). Genome Haploidisation with Chromosome 7 Retention in Oncocytic Follicular Thyroid Carcinoma. PLoS ONE.

[31] Doerfler W.R., Nikitski A.V., Morariu E.M., Ohori N.P., Chiosea S.I., Landau M.S., Nikiforova M.N., Nikiforov Y.E., Yip L., Manroa P. (2021). Molecular Alterations in Hürthle Cell Nodules and Preoperative Cancer Risk. Endocr. -Relat. Cancer.

[32] Van der Tuin K., de Kock L., Kamping E.J., Hannema S.E., Pouwels M.-J.M., Niedziela M., van Wezel T., Hes F.J., Jongmans M.C., Foulkes W.D. (2019). Clinical and Molecular Characteristics May Alter Treatment Strategies of Thyroid Malignancies in DICER1 Syndrome. J. Clin. Endocrinol. Metab..

[33] Gubbiotti M.A., Andrianus S., Baloch Z. (2023). THADA-IGF2BP3 Fusions Detected in Fine-Needle Aspiration Specimens of Thyroid Nodules: An Institutional Experience. Diagn. Cytopathol..

[34] Morariu E.M., McCoy K.L., Chiosea S.I., Nikitski A.V., Manroa P., Nikiforova M.N., Nikiforov Y.E. (2021). Clinicopathologic Characteristics of Thyroid Nodules Positive for the THADA-IGF2BP3 Fusion on Preoperative Molecular Analysis. Thyroid.

[35] Ganly I., Ricarte Filho J., Eng S., Ghossein R., Morris L.G.T., Liang Y., Socci N., Kannan K., Mo Q., Fagin J.A. (2013). Genomic Dissection of Hurthle Cell Carcinoma Reveals a Unique Class of Thyroid Malignancy. J. Clin. Endocrinol. Metab..

